# Ubiquitination-Mediated Inflammasome Activation during Bacterial Infection

**DOI:** 10.3390/ijms20092110

**Published:** 2019-04-29

**Authors:** Tao Xu, Yu Guo, Xiaopeng Qi

**Affiliations:** 1 Key Laboratory of Animal Models and Human Disease Mechanisms of Chinese Academy of Sciences/Key Laboratory of Bioactive Peptides of Yunnan Province, Kunming Institute of Zoology, Chinese Academy of Sciences, Kunming 650223, China; xutao@mail.kiz.ac.cn (T.X.); guoyu@mail.kiz.ac.cn (Y.G.); 2 KIZ-CUHK Joint Laboratory of Bioresources and Molecular Research in Common Diseases, Kunming Institute of Zoology, Chinese Academy of Sciences, Kunming 650223, China

**Keywords:** inflammasome, ubiquitination, bacterial infection, immune response

## Abstract

Inflammasome activation is essential for host immune responses during pathogenic infection and sterile signals insult, whereas excessive activation is injurious. Thus, inflammasome activation is tightly regulated at multiple layers. Ubiquitination is an important post-translational modification for orchestrating inflammatory immune responses during pathogenic infection, and a major target hijacked by pathogenic bacteria for promoting their survival and proliferation. This review summarizes recent insights into distinct mechanisms of the inflammasome activation and ubiquitination process triggered by bacterial infection. We discuss the complex regulatory of inflammasome activation mediated by ubiquitination machinery during bacterial infection, and provide therapeutic approaches for specifically targeting aberrant inflammasome activation.

## 1. Introduction

Early innate immune responses play essential roles in host defense against bacterial infection and rely on the recognition of pattern recognition receptors (PRRs) [1]. PRRs can be divided into two major classes including membrane-bound Toll-like receptors (TLRs) and C-type lectin receptors (CLRs), and non-membrane bound intracellular receptors such as the RIG-I-like receptor (RLR), AIM2-like receptor (ALR), and nucleotide-binding protein domain and leucine-rich repeat-containing (NLR) proteins [2]. AIM2 and a subgroup of the NLR proteins have the ability to assemble inflammasomes [3,4]. Inflammasomes are multiple protein complexes activated in response to infections or sterile stress, and involved in the activation of pyroptotic cell death and release of proinflammatory cytokines IL-1β and IL-18 [5,6]. Dysregulation of the inflammasome activation can result in a wide range of infectious and inflammatory diseases, cancer, metabolic and autoimmune disorders [7,8]. Both extracellular and intracellular bacterial infections trigger different types of inflammasome activation through engagements with distinct inflammasome sensors [5]. The inflammasome process is tightly regulated and recent studies have significantly advanced our understanding on the regulation of inflammasome activation, assembly and posttranslational modification of inflammasome proteins such as ubiquitin and ubiquitin-like modifications [5,9,10,11,12,13]. Ubiquitination is a highly complex and dynamic post-translational protein modification that is involved in targeting protein for proteasomal degradation, interaction and signaling. Ubiquitination has multiple effects in the bacteria-host interface and bacteria also can target host-cell ubiquitin and ubiquitin-like pathways for invasion [14]. In this review, we highlight new observations on bacterial infection triggering inflammasome activation and ubiquitination, as well as ubiquitination-mediated inflammasome activation.

## 2. Bacterial Infection Triggers Inflammasome Activation

Inflammasome activation plays essential roles in the innate immune defense against bacterial infection. The inflammasome-mediated proinflammatory cytokine production and pyroptotic cell death contribute to the induction of inflammatory response and host defense. Assembly of inflammasome initiates from the engagement of bacterial pathogen associated molecular patterns (PAMPs) and NLRs or ALRs, such as NLR family pyrin domain containing 1 (NLRP1), NLR family pyrin domain containing 3 (NLRP3), NLR family CARD domain-containing protein 4 (NLRC4), AIM2, and Pyrin receptors [15,16].

NLRP1 also known as NACHT leucine-rich-repeat protein 1(NALP1), was the first identified NLR family member to form inflammasome complex mediating caspase-1 activation and IL-1β maturation in 2002 [17]. Until ten years later, NLRP1 activation was identified to be regulated by the anthrax lethal toxin (LT), and LT-mediated NLRP1 cleavage was required and sufficient for its activation [18,19,20]. The NLRP1 inflammasome activation can occur independently of the adaptor apoptosis-associated speck-like protein containing a CARD (ASC)-dependent caspase-1 autoproteolysis and speck formation [21]. *Bacillus anthracis* infection also can trigger caspase 1 activation through the NOD2-NLRP1 complex formation induced by bacterial muramyl dipeptide (MDP) [22]. In NOD2-mediated Crohn’s disease (CD), the NLRP1 and NLRP3 inflammasome activation is essential for the distinct outcomes of decreased inflammatory cytokines but increased bacterial killing activity [23]. Furthermore, the MDP induced NLRP1 inflammasome formation and caspase-1 activation was demonstrated to be suppressed by Bcl-2 and Bcl-XL binding [24]. NLRP1 activation in macrophages and dendritic cells triggered pyroptotic cell death and inflammatory cytokine release confer to the resistance to *B. anthracis* infection [25]. Instead, the NLRP1 activation in hematopoietic progenitor cells results in cytopenia and immunosuppression, and the deficiency of NLRP1 promotes recovery from the lymphocytic choriomeningitis virus (LCMV) infection and chemotherapy [26].

NLRP3 (also known as NALP3) inflammasome is the most extensively studied inflammasome and can be activated by a broad range of bacterial pathogens, yet the cellular mechanisms that regulate the NLRP3 inflammasome activation is not completely understood [27]. NLRP3 inflammasome components comprise of NLRP3, ASC, and caspase-1. Two signals are required for the NLRP3 inflammasome activation. The first signal mediates transcription of NLRP3 and IL-1β through NF-κB signaling, and the second signal triggers inflammasome assembly and capase-1 activation through exposure to endogenous and exogenous danger molecules, such as K^+^ efflux, ATP, ROS signal, lysosomal rupture, pore-forming toxins, and other microbial virulence factors [28,29]. Extensive studies were performed to investigate the mechanism of NLRP3 inflammasome assembly, and growing evidences indicate that the mitochondria localization of NLRP3 inflammasome components is essential for its activation. Response to activation, NLRP3 and ASC were reported to co-localize with endoplasmic reticulum (ER) and mitochondria, and mitochondria produced ROS activated the NLRP3 inflammasome [30]. It was shown that the microtube mediated the mitochondria transport and apposition of ASC on mitochondria to NLRP3 on the ER [31]. Furthermore, the mitochondria-associated adaptor molecule, MAVS, was defined to mediate the NLRP3 recruitment to mitochondria and subsequent events [32]. Recent studies also demonstrated that newly synthesized mitochondrial DNA (mtDNA) was necessary for the NLRP3 inflammasome activation [33], and the trans-Golgi network (TGN) served as a scaffold for NLRP3 aggregation puncta leading to ASC polymerization mediated by phosphatidylinositol-4-phosphate (PtdIns4P) on dispersed TGN [34].

NLRP3 inflammasome plays dual protective and pathogenic roles in host defense against bacterial infection due to the caspase-1-dependent cell death and released inflammatory cytokines that contribute to limit the spread of invading pathogens and simultaneously are involved in various inflammatory pathologies [35,36]. Various bacterial components can induce the NLRP3 inflammasome activation through diverse mechanisms. Pore-forming toxins and bacterial RNAs represent the major triggers of the NLRP3 inflammasome activation. Pore-forming toxin secreted by bacteria disrupt plasma membrane permeability that allow the efflux of intracellular potassium and trigger the NLRP3 inflammasome activation [28,37]. NLRP3 also recognizes microbial components and plays an essential role in the inflammasome activation, such as human NLRP3 inflammasome senses all three types of bacterial RNAs-mRNA, tRNA, and rRNAs [38]. Gram-negative bacteria induced NLRP3 inflammasome activation was identified as TRIF and caspase-11 dependent, and caspase-11 mediated non-canonical inflammasome activation leading to pyroptotic cell death instead of processing of IL-1β and IL-18 through engagement with intracellular LPS [39,40,41,42]. Recently, Rathinam et al. demonstrated that bacterial outer membrane vesicles derived from gram-negative bacteria were essential for LPS delivery to host the cell cytosol and caspase-11 activation [43]. Furthermore, Broz group showed that host GBPs recruited to the intracellular LPS-containing OMVs and contributed to the LPS triggered non-canonical caspase-11 activation [44]. NLRP3 inflammasome activation was also reported to be negatively regulated in the transcriptional level and assembly process by various molecules, such as TRIM 30, A20, nitric oxide, carbon monoxide, and aryl hydrocarbon receptor (AhR) [45,46].

NLRC4 was initially identified as an adaptor protein mediating inflammasome activation specially responsive to the intracellular bacteria *Salmonella* typhimurium [47]. Later on, it was shown that bacterial flagellin and type III secretion system (T3SS) proteins rod and needle engagements with NLR family, apoptosis inhibitory protein (NAIP) proteins triggered the NLRC4 inflammasome activation [48,49,50]. Biochemical and genetic functional studies have revealed that NAIP1, NAIP2 and NAIP5 were cytosolic receptors that directly recognize bacterial Needle, Rod and Flagellin proteins to trigger the NLRC4 inflammasome activation [51,52]. Thus, the NAIP-NLRC4 inflammasome plays a key role in host defense against bacterial infection through sensing flagellin and T3SS rod/needle proteins in the bacteria [53]. Recently, IRF8 was demonstrated to mediate the transcription of *Naip* genes (*Naip1*, *Naip2*, *Naip5*, and *Naip6*) and play key roles for the NLRC4 inflammasome activation [54]. NLRC4 phosphorylation at Ser533 mediated by LRRK2 was crucial for the NLRC4 inflammasome activation during host defense against the *Salmonella* typhimurium infection [55]. Extensive studies of the *L. pneumophila* infection model revealed the protective role of the NAIP5–NLRC4 inflammasome activation through the development of a coordinated host response [53]. In addition, the NLRC4 activation and caspase-1-indcued cell death instead of proinflammatory cytokines act as innate immune effector mechanism against intracellular bacteria *Salmonella* typhimurium through exposing intracellular bacteria to killing by ROS in neutrophils [56].

Transfection of bacterial and host DNA was reported to trigger the NLRP3-independent and ASC-dependent inflammasome activation in macrophages [57], indicating that a novel intracellular receptor mediates cytosolic DNA-induced inflammasome activation. In 2009, AIM2 was demonstrated to act as a cytosolic DNA sensor that forms inflammasome with ASC and caspase-1 independently by four groups [58,59,60,61]. In contrast to the NLRP3 and NLRC4 inflammasomes, the AIM2 inflammasome is rarely investigated. In the context of bacterial infection, AIM2 is recognized as mainly responsive to the *Francisella* infection and partially involved in the infection with *Mycobacteria* and *Listeria* [62,63,64,65,66,67]. The observation of the AIM2 inflammasome activation triggered by the cytosolic DNA exposed by intracellular bacteria provides a promising tool to study the intracellular bacteriolysis. Type I interferon signaling was identified essential for the *Francisella* infection-induced inflammasome activation by Monack group, whereas which inflammasome involved was not known at that time [68]. Now we know that type I IFN signaling induces IRF1 expression, which in turn activates the expression of GBP proteins and IRGB10 during the *Francisella* infection; IRGB10 and GBPs coordinate the bacteriolysis of the *Francisella* and inflammasome activation [69,70,71]. Structural analysis revealed that DNA serves as an oligomerization platform for the AIM2 inflammasome and the length of DNA not the sequence determines the activation of the AIM2 inflammasome [72]. Whether the host DNA also contributes to the activation of the AIM2 inflammasome during bacterial infection remains unclear.

Pyrin protein is encoded by the *MEFV* gene in human, whose mutation is associated with human autoinflammatory disease known as familial Mediterranean fever (FMF) [73,74]. Gain-of-function Pyrin induces constitutively activation of caspase-1, which is the ASC-dependent NLRP3-independent inflammasome activation [75]. The pyrin inflammasome can also be activated by the intracellular *Burkholderia cenocepacia* infection, whereas the physiological function of Pyrin remains unknown [76]. In 2014, Shao group elucidated the components of the Pyrin inflammasome and activation mechanism in response to various bacterial cytotoxins stimulation [77]. They identified that inactivation of Rho GTPase is the direct trigger of the Pyrin inflammasome, and the Rho-inactivating toxins and *Burkholderia cenocepacia* all have the capacity to inactivate Rho family members and trigger the Pyrin inflammasome activation [77]. Subsequently, it was demonstrated that phosphorylation of Pyrin, 14-3-3 protein binding, and microtubule dynamics are critical for modulating the Pyrin inflammasome activation [78,79,80]. In addition, pathogenic *Yersinia pestis* can secret multiple effectors known as YoPs to trigger and inhibit the Pyrin inflammasome activation. For example, both YopE and YopT effectors trigger the Pyrin inflammasome through inactivating RhoA, while YopM can hijack host kinase PRKs to regulate phosphorylation of 14-3-3 binding sites of Pyrin protein, which lead to inhibition of the Pyrin inflammasome [81,82]. Different from other defined inflammasomes, the Pyrin inflammasome does not directly recognize molecular patterns, but rather relies on the inactivation of Rho GTPase and the disturbances in cytoplasmic homeostasis need to be further examined [83].

One of the biggest challenges in the inflammasome field was how inflammatory caspases caspase-1 and caspase4/11 caused pyroptotic cell death. In 2015, Shao group and Dixit group independently identified Gasdermin D (GSDMD) as an executioner of the pyroptosis downstream of canonical inflammasome caspase-1 and non-canonical inflammasome caspase-11/4/5 [84,85]. They both found that the N-terminal of GSDMD cleaved by inflammatory caspases was required and sufficient for pyroptotic cell death. Later on, these two groups both reported that the pore-forming activity of GSDMD compromises the integrity of the membrane [86,87]. The Shao group further demonstrated that other gasdermin-N domains, GSDMA3 and GSDMA, also formed pores within the membrane, and most gasdermin pores contained 16 symmetric protomers with an inner diameter of 10–14 nm [86].

The identification of GSDMD and its pore-forming activity provided significant advances in our understanding of substrates of inflammatory caspases and pyroptosis [88,89]. Recently, the events downstream of GSDMD has been revealed by Petr Broz group, they found that the ESCRT-dependent membrane repair triggered by calcium influx signaling through GSDMD pores negatively regulates pyroptosis, and the inhibition of the ESCRT-III machinery enhances pyroptosis and IL-1β secretion [90]. Up-to-date, GSDMD-mediated pyroptosis has been extensively studied in molecular mechanism and various disease models. GSDMD-mediated pyroptosis was identified critical for the systemic autoinflammatory pathology in Familial Mediterranean Fever knock-in mice (*Mefv*^*V726A*/*V726A*^) [91]. In the context of bacterial infection, GSDMD is essential for the *F. novicida*-triggered AIM2 inflammasome activation, the deficiency of GSDMD increases the mouse susceptibility to the *F. novicida* infection [92]. Instead, GSDMD-mediated neutrophil death dampens the bactericidal activity against the *E. coli* infection, and the delayed neutrophil death in GSDMD-deficient mice results in enhanced host defense [93]. Furthermore, GSDMD is also required for the *Brucella abortus* infection-triggered caspase-11-mediated pyroptosis and host defense [94].

## 3. Inflammasome Activation and Ubiquitination

Precise regulation of the inflammasome activation is critical for adequate immune responses while limiting tissue damage and preventing autoimmunity. Emerging studies have shown that post-translational modifications (PTMs) of inflammasome components are crucial for the inflammasome activation, such as phosphorylation, ubiquitination, S-Nitrosylation, ADP-ribosylation and proteolysis process, and PTMs of inflammasome components have been recognized as a major regulatory mechanism for the inflammasome activation [95]. Ubiquitination is a post-translational modification by the covalent attachment of a small protein ubiquitin that governs a variety of eukaryotic cellular processes and serves as an important signal in the host immune responses to the pathogenic infection. In eukaryotes, the conjugation of ubiquitin to target proteins is sequentially mediated by the ubiquitin-activating enzyme E1, the ubiquitin-conjugating enzyme E2 and the ubiquitin ligase E3 [96]. Initially, ubiquitination was reported to mediate the delivery of inflammasome to autophagosomes for destruction through the recruitment of autophagic adaptor p62 and ASC polyubiquitination [97]. Recently, increasing evidences indicate that ubiquitination mediates inflammasome assembly and activation. We will describe the roles of ubiquitination of ASC, NLRP3 and other inflammasome sensors and components in the inflammasome activation.

The NLRP3 inflammasome is the best characterized for post translational regulations, because various pathogenic infections and host-derived damage-associated molecular patterns can trigger its activation [10]. Alnemri et al. first demonstrated that the deubiquitination of NLRP3 was required for the NLRP3 inflammasome activation by using deubiquitinase inhibitors PR-619 and WP1130 [98]. Yuan group first demonstrated that the NLRP3 deubiquitination mediated by deubiquitinase BRCC3 was essential for its activation [99]. They found the inhibition of deubiquitination by G5 triggered the NLRP3 ubiquitination and inhibited NLRP3 inflammasome activation. Through the screening library of deubiquitinases and functional analysis, they defined that the deubiquitinase BRCC3 can promote the NLRP3 inflammasome activation through deubiquitinating NLRP3. This study demonstrated that the NLRP3 deubiquitination played critical roles for its assembly and activation, even without knowing the detailed ubiquitination process. Lopez-Castejon et al. also reported that small molecule inhibitors of the deubiquitinase enzyme activity inhibited the inflammasome activation through impairing the ASC oligomerization [100]. In addition, a study from Alnemri group revealed that signaling through the TLR4 recognition induced the mitochondrial reactive oxygen species production, which in turn promoted the NLRP3 deubiquitination and its activation [98]. Regarding the mechanism by which deubiquitination mediates the inflammasome assembly and activation, the David Brough group recently identified that deubiquitinases USP7 and USP47 regulate the NLRP3 inflammasome activation by promoting ASC oligomerization and speck formation [101]. Lee et al. also reported that deubiquitinase USP50 interacted with ASC and removed the K63-linked ubiquitin of ASC to mediate the ASC oligomerization and NLRP3 inflammasome activation [102].

Met1-linked linear ubiquitin modification formed by the linear ubiquitination assembly complex (LUBAC) has been reported to regulate the NF-κB activation by the linear polyubiquitylation of NF-kappa-B essential modulator (NEMO) [103]. In 2014, Rodgers et al. reported that the LUBAC-mediated ASC linear ubiquitination was essential for the assembly of the NLRP3/ASC complex and NLRP3 inflammasome activation [104,105]. So far, numbers of E3 ligase-regulated NLRP3 inflammasome activation have been reported. These E3 ligases exhibited either positive or negative roles for the NLRP3 inflammasome activation depending on the ubiquitin linkage types and targets. Zhou group reported that the E3 ligase MARCH7 promoted K48-linked polyubiquitination of the NLRP3 and NLRP3 degradation, which contributed to the dopamine inhibited NLRP3 inflammasome activation and neuroinflammation [106]. TRIM31, Cullin1 and ARIH2 also were demonstrated to negatively regulate the NLRP3 inflammasome activation through the ubiquitination of NLRP3 [107,108,109]. In contrast, TRAF3 and Pellino2 were reported to promote the NLRP3 inflammasome activation via mediating K63-linked polyubiquitination of ASC and NLRP3, respectively [110,111]. TRAF6 was also required for the NLRP3 inflammasome activation through mediating the NLRP3 oligomerization and ubiquitination [112]. F-box containing the E3 ubiquitin ligase FBXL2 was demonstrated to mediate the NLRP3 ubiquitination and proteasomal degradation, LPS priming inhibited the FBXL2 activity and thus promoted the NLRP3 stability [113]. In addition, the phosphorylation of NLRP3 also has an effect on its ubiquitin modification. Guo et al. reported that bile acids inhibited inflammation and exhibited improvement in metabolic syndromes through directly inhibiting the NLRP3 inflammasome activation [114]. Mechanistically, the bile acids treatment induces the PKA activation and NLRP3 phosphorylation at Ser 291, which drives the NLRP3 ubiquitination without knowing the E3 ligase involved.

How the NLRP1B cleavage induced by LT resulting in the NLRP1B inflammasome activation is an unsolved puzzle. The Vance group first showed that the proteasome-mediated degradation of NLRP1B N-terminal domains was necessary and sufficient for the NLRP1B activation. In addition to LT, they also identified that *Shigella flexneri* E3 ubiquitin ligase IpaH7.8 regulated the NLRP1B ubiquitination, degradation and activation [115]. Simultaneously, the Bachovchin group used genome-wide screening and identified that the N-end rule protein E3 ligases UBR2/4 mediated the N-end rule proteasomal degradation of NLRP1B and its activation [115,116]. Shao group also demonstrated the UBR2 mediated ubiquitination and N-terminal cleavage of NLRP1B through interacting with the ubiquitin conjugating enzyme E2O [117]. These studies demonstrated ubiquitination and the N-terminal degradation were essential for NLRP1B activation.

Bacteria E3 ligases can subvert the host immune response by targeting inflammasome components. *Shigella* IpaH7.8 is one of best studied bacterial E3 ligases. Suzuki et al. demonstrated that *Shigella* IpaH7.8 E3 ubiquitin ligase mediated ubiquitination of GLMN for proteasomal degradation and promoted both the NLRP3 and NLRC4 inflammasome activation without knowing the detailed mechanism [118]. Further, Wei et al. reported that the Yersinia Type III secretion effector YopM interacted with NLRP3 and mediated its K63-linked polyubiquitination, which was crucial for the NLRP3-mediated cell death [119]. Yen et al. showed that the effector protein NleA of severe gastrointestinal disease-inducing pathogens enteropathogenic could subdue IL-1β secretion through inhibiting caspase-1 activation [120]. The authors identified NLRP3 as one target of NleA and the interaction of NleA and NLRP3 blocked the deubiquitination of NLRP3 resulting in the decreased NLRP3 inflammasome activation.

E3 ligase TRIM11 was reported to interact with AIM2 and induce its degradation through the recruitment of p62 for autophagy-dependent degradation but not ubiquitination-mediated proteasome degradation [121]. In contrast to the NLRP3 ubiquitination, the ubiquitination of AIM2 was less studied. Type I IFN signaling and its inducible GBP proteins were illustrated to mediate the AIM2 inflammasome activation [70,71]. Bacterial ubiquitin ligase IpaH9.8 secreted into the cell cytosol caused the ubiquitination of GBP proteins and their proteasome degradation [122,123], which highlighted the importance of ubiquitination in the regulation of the AIM2 inflammasome activation and host defense. In addition to the caspase-1-dependent pyroptosis, the ubiquitinated NLRC4 regulated by SUG1 was demonstrated to mediate FADD-caspase-8 dependent apoptosis. NLRC4 point mutation NLRC4^H433P^ enhanced interaction with SUG1 which mediated the NLRC4^H433P^ ubiquitination and interaction with FADD leading to the caspase-8-dependent cell death [124]. Instead, the direct modification of the Pyrin ubiquitination has not been reported yet.

Regarding the ubiquitination of caspase-1 and its targets, the E2 conjugate enzyme UBE2L3 was shown to be one of caspase-1 targets and mediates IL-1β proteasome degradation through K48-linked polyubiquitination [125]. The NF-κB inhibitor A20 is a deubiquitinase, it was investigated to prevent inflammatory responses through regulating the K63-linked ubiquitination of pro-IL-1β, and its associated complex. The deficiency of A20 causes a spontaneous NLRP3 inflammasome activation response to the LPS treatment alone [126]. Inhibitors of apoptosis cIAP1 and cIAP2 were reported to regulate K63-linked ubiquitination of caspase-1 in the complex with TRAF2, and mediate the caspase-1 activation [127,128]. Caspase-1 was also shown to be modified by the ubiquitin-like protein Nedd8, which directly interacted with caspase-1 and promoted the caspase-1 activation [129].

In summary, recent studies have provided significant advances of the inflammasome activation regulated by ubiquitination. So far, ubiquitination events were almost identified in every inflammasome components and even the upstream and downstream molecules of the inflammasome activation [12]. The reported inflammasome activation regulated by ubiquitination were summarized in Table 1.

## 4. Bacterial Infection and Ubiquitination-Mediated Immune Responses

During bacterial infection, the components of host ubiquitination machinery can be regulated or exploited by a variety of bacterial effector proteins. Bacterial pathogens use multiple strategies to exploit the host ubiquitination and deubiquitination pathways to favor their survival and growth [130]. To subvert host ubiquitin machinery, bacterial pathogens have evolved different classes of enzymes to mimic specific components of the host ubiquitin system such as RING, HECT, novel E3 ligases (NELs), non-E3 ligase enzymes and bacterial deubiquitinating enzymes [131]. Bacterial effector SopA from the *Salmonella* type III secretion system was the first identified as HECT-type E3 ligase [132], which targeted and mediated the ubiquitination of two host E3 ligases TRIM56 and TRIM65 for promoting type I IFN production and innate immune responses [133]. Another well studied bacterial HECT-type E3 ligase was NleL derived from *E. coli*, which catalyzed the K6- and k48-linked polyubiquitin formation through a thioester intermediate with ubiquitin [134]. NleG type III effectors of pathogenic *E. coli* were recognized as RING/U-box E3 ubiquitin ligases, bacterial NlgG effectors displayed strong autoubiquitination activity and were demonstrated to interact with human E2 enzymes for supporting the activity [135]. Multiple effector proteins from *Legionella pneumophila* have been defined as the U-box and F-box domain-containing E3 ligases. LubX and GobX were two U-box domain-containing effectors to mediate the ubiquitination of other bacterial effectors and host cellular proteins. *L. pneumophila* F-box effectors LegU1 and AnkB were reported to direct the ubiquitination of the host chaperone protein BAT3 and recruit polyubiquitinated proteins into *Legionella* containing vacuole for promoting the *L. pneumophila* intracellular replication, respectively [136,137].

In addition to the conventional ubiquitination process, pathogenic bacteria evolved unconventional NELs and non-E3 ligase enzymes through an E1- and E2-independent process. Numbers of NELs have been discovered in *Salmonella* and *Shigella* pathogens, such as SspH2 of *Salmonella* and IpaH family proteins from *Shigella* [138,139]. The host NF-κB and inflammasome pathways were demonstrated as major targets of *Shigella* IpaH effectors. In addition to the inflammasome activation mediated by bacterial NELs described above, bacterial novel E3 ubiquitin ligases IpaH9.8 and IpaH0722 were reported to target NEMO and TRAF2 for the ubiquitination-mediated proteasome degradation and dampen NF-κB-dependent inflammatory responses [140,141]. IpaH1.4 and IpaH2.5 interacted with LUBAC subunits and conjugated the K48-linked ubiquitin to HOIP for proteasomal degradation of HOIP and inhibiting of NF-κB signaling [142]. Effector IpaH4.5 was reported to target TBK1 for mediating its K48-linked polyubiquitination and proteasome degradation, which inhibited the IRF3 activation and antibacterial responses [143]. One of the significant advances in the understanding of bacterial effectors hijack of host cell ubiquitin machinery is the identified non E3 ligase enzymes that catalyze ubiquitylation in an E1- and E2-independent manner. Qiu et al. first reported the *L. pneumophila* effector protein SidE family ubiquitinated host Rab small GTPases without involvement of the E1 and E2 enzymes [144]. In fact, the mono-ADP-ribosyltransferase (mART) motif within the SidE family member SdeA was found to catalyze the ADP-ribosylated ubiquitin on Arg42 of ubiquitin. Nucleotidase-phosphohydrolase domain (NP) mediates the cleavage of phosphodiester bond in the ADP-ribosylated ubiquitin creating a phosphor-ribosylated ubiquitin, which is conjugated to substrate proteins. Subsequently, Ivan Dikic group demonstrated that the phosphoribosylated ubiquitin promoted substrate proteins serine ubiquitination and prevented the activation of E1 and E2 enzymes within conventional ubiquitination cascade [145]. In addition, Ralph R. Isberg group identified Sde promoted the ubiquitination of Rtn4 with phosphor-ribosylated ubiquitin resulting in the rearrangement of tubular ER for replication [146].

Given that ubiquitination is a reversible process, bacterial pathogens also evolved effectors mimicking host DUBs to remove ubiquitin from substrate proteins. The non E3 ligase enzyme SidE was initially recognized as deubiquitinase with preference for the removal of K63-linked ubiquitin from the phagosomal surface [147]. Another well-studied bacterial deubiquitinase is the SseL from *Salmonella*. Holden et al. identified SseL as deubiquitinase for hydrolyzing mono- and polyubiquitin proteins with a preference of K63-linked ubiquitin chains, which led to the lower autophagic flux and favor intracellular *Salmonella* replication [148,149]. Collectively, these studies suggest that ubiquitination and deubiquitination pathways are indeed important targets by bacterial pathogens exploitation to manipulate host immune responses and promote their survival and replication.

## 5. Concluding Remarks and Perspectives

Based on the work reviewed above and elsewhere [11,12,130], the inflammasome activation and ubiquitination process are two major events of host immune responses during bacterial infection. Similar to the ubiquitination mediated NF-κB and type I IFN signaling pathways, the ubiquitination-mediated inflammasome activation was increasingly identified, which added an additional layer of complexity in the regulation of the inflammasome activation. Inflammasome activation is a double-edged sword for intracellular bacterial infection, it triggers an inflammatory response and simultaneously, the replication niche of intracellular bacteria is reduced due to the pyroptotic cell death. Thus, the activation of inflammasome needs to be tightly regulated at multiple layers during bacterial infection. Ubiquitination is a reversible process and inhibitors that regulate ubiquitin machinery are an attractive therapeutic approach for specifically targeting the aberrant inflammasome activation.

Ubiquitination could regulate the components of the inflammasome complex and the regulators that are involved in the inflammasome activation. Our current understanding of the interplay between the ubiquitination and inflammasome activation during bacterial infection is still limited. Several important questions need to be addressed. The detailed mechanisms by which deubiquitinates mediate the ASC oligomerization are not clear. What are the E3 ligases that mediate the K63-linked ubiquitination of ASC? Do bacterial E3 ligases or non E3 ligase enzymes directly modify components of host cellular inflammasome and mediate the inflammasome activation? How is the ubiquitination activity regulated by bacterial infection? Overall, the ubiquitination as a regulatory mechanism for the inflammasome activation will remain a perspective and interesting area of study for the future.

## Figures and Tables

**Table 1:** Known ubiquitination-mediated inflammasome activation.

Modification Type	Target	Enzyme	Function (Ref)
Deubiquitination	**NLRP3**	NA	Deubiquitination of NLRP3 is required for its activation [98]
Deubiquitination	**NLRP3**	**BRCC3**	Deubiquitination of NLRP3 by BRCC3 promotes inflammasome activation [99]
Ubiquitination (K48)	**NLRP3**	**MARCH7**	MARCH7 mediates K48-linked polyubiquitination of NLRP3 and proteasome degradation [106]
Ubiquitination (K48/K63)	**NLRP3**	**ARIH2**	ARIH2 mediates K48- and K63-linked polyubiquitination of NLRP3 and negatively regulates NLRP3 inflammasome activation [108]
Ubiquitination	**NLRP3**	**TRAF6**	The ubiquitin E3 ligase activity of TRAF6 mediates NLRP3 oligomerization and inflammasome activation [112]
Ubiquitination (K63)	**NLRP3**	**Pellino2**	Pellino2 mediates K63-linked ubiquitination and activation of NLRP3 [110]
Ubiquitination (K48)	**NLRP3**	**TRIM31**	TRIM31 mediates K48-linked polyubiquitination of NLRP3 and inhibits NLRP3 inflammasome activation [107]
Ubiquitination	**NLRP3**	**Cullin1**	Cullin1 mediates NLRP3 ubiquitination and inhibits NLRP3 inflammasome activation [109]
Ubiquitination (K63)	**NLRP3**	**YopM**	Bacterial E3 ligase YopM mediates K63-linked polyubiquitination of NLRP3 and induces NLRP3-mediated cell death [119]
Deubiquitination	**NLRP3**	**NleA**	Bacterial effector NleA binds to NLRP3 and inhibits deubiquitination and activation of NLRP3 [120]
Ubiquitination	**NLRP3 (Lys689)**	**FBXL2**	FBXL2 mediates ubiquitination and degradation of NLRP3 [113]
Ubiquitination	**NLRP3**	NA	PKA induces phosphorylation of NLRP3 which results in NLRP3 ubiquitination and degradation [114]
Ubiquitination	**GLMN-NLRP3/NLRC4**	**IpaH7.8**	Bacterial E3 ligase IpaH7.8 mediates ubiquitination of GLMN, an inhibitor for the NLRP3 and NLRC4 inflammasomes, and induces inflammasome activation [118]
Linear ubiquitination	**ASC**	**LUBAC**	Linear ubiquitination of ASC is required for NLRP3/ASC assembly and activation [104]
Ubiquitination	**ASC**	NA	Autophagy induced by inflammasome signals targets polyubiquitinized ASC for degradation [97]
Deubiquitination	**ASC**	NA	Deubiquitination inhibition blocks ASC oligomerization and inflammasome activation [100]
Deubiquitination	**ASC and NLRP3**	**USP7** and **USP47**	USP7 and USP47 mediate deubiquitination of ASC and NLRP3, which promotes ASC oligomerization and NLRP3 inflammasome activation [101]
Deubiquitination (K63)	**ASC**	**USP50**	USP50 binds to ASC and directly deubiquitinates K63-linked polyubiquitination of ASC, which is required for ASC oligomerization and inflammasome activation [102]
Ubiquitination (K63)	**ASC (Lys174)**	**TRAF3**	MAVS recruits TRAF3 to mediate K63-linked ubiquitination of ASC and promotes inflammasome activation [111]
Ubiquitination and N-terminal degradation	**NLRP1B**	NA or **IpaH7.8**	Anthrax lethal toxin cleavage results in proteasome-mediated degradation of the N-terminal domains of NLRP1B and NLRP1B activation; Bacterial ubiquitin ligase IpaH7.8 degrades and activates NLRP1B [115]
Ubiquitination and N-terminal degradation	**NLRP1B**	**UBR2/4**	UBR2/4 mediates ubiquitination of N-terminal domains of NLRP1B and proteasome degradation, which results in NLRP1B activation [116]
Ubiquitination and N-terminal degradation	**NLRP1B**	**UBR2** and **E2O**	E3 ligase UBR2 and E2 conjugating enzyme E2O mediate ubiquitination and degradation of N-terminal domains of NLRP1B and NLRP1B activation [117]
Ubiquitin (p62 recruitment)	**AIM2**	**TRIM 11**	TRIM11 undergoes autoubiquitination and recruits AIM2 to p62 for degradation, which inhibits AIM2 inflammasome activation [121]
Ubiquitination	**NLRC4(H443P)**	**SUG1**-NA	SUG1 interacts with NLRC4 mutant NLRC4(H443P) and causes Caspase-8 activation and cell death [124]
Ubiquitination (K63)	**Caspase-1**	**cIAP1**, **cIAP2**, **TRAF2**	cIAP1, cIAP2, and TRAF2 mediate K63-linked polyubiquitination and activation of Caspase-1 [127]
Ubiquitination (K48)	**IL-1β**	**BUBE2L3**	Caspas-1 target UBE2L3 mediates K48-linked polyubiquitination of pro-IL-1β and degradation [125]
Deubiquitination (K63)	**IL-1β (Lys133)**	**A20**	A20 deubiquitinates IL-1β and suppresses spontaneous NLRP3 inflammasome activation [126]
